# Oxytocin Controls Chondrogenesis and Correlates with Osteoarthritis

**DOI:** 10.3390/ijms21113966

**Published:** 2020-05-31

**Authors:** Christian H. Roux, Didier F. Pisani, Pierre Gillet, Eric Fontas, Hédi Ben Yahia, Mansour Djedaini, Damien Ambrosetti, Jean-François Michiels, Patricia Panaia-Ferrari, Véronique Breuil, Astrid Pinzano, Ez-Zoubir Amri

**Affiliations:** 1 Université Côte d’Azur, French National Centre for Scientific Research (CNRS), Inserm, iBV, 06107 Nice, France; benyahia@unice.fr (H.B.Y.); Djedaini@unice.fr (M.D.); 2 Department of Rheumatology, Nice University Hospital, Pasteur Hospital, 06003 Nice, France; breuil.v@chu-nice.fr; 3 Université Côte d’Azur, CNRS, LP2M, F-06003 Nice, France; pisani@nice.fr; 4 UMR 7365 French National Centre for Scientific Research (CNRS)–Université de Lorraine, ‘Ingénierie Moléculaire et Physiopathologie Articulaire’ (IMoPA), F54505 Vandoeuvre-lès-Nancy, France; pierre.gillet@univ-lorraine.fr (P.G.); astrid.pinzano@univ-lorraine.fr (A.P.); 5 Department of Clinical Research, Nice University Hospital, Cimiez Hospital, F-06003 Nice, France; fontas.e@chu-nice.fr; 6 Université Côte d’Azur, UFR Médecine, F-06107 Nice, France; Ambrosetti.d@chu-nice.fr (D.A.); michiels.jf@chu-nice.fr (J.-F.M.); 7 Anatomopathology Service, Pasteur Hospital, Centre Hospitalier Universitaire de Nice, F-06003 Nice, France; 8 Clinical Chemistry Laboratory, University Hospital of Nice, F-06003 Nice, France; Panaia-Ferrari.p@chu-nice.fr

**Keywords:** osteoarthritis, chondrocyte, hMADS cells, oxytocin, clinical OA severity

## Abstract

This study investigated the relationship of oxytocin (OT) to chondrogenesis and osteoarthritis (OA). Human bone marrow and multipotent adipose-derived stem cells were cultured in vitro in the absence or presence of OT and assayed for mRNA transcript expression along with histological and immunohistochemical analyses. To study the effects of OT in OA in vivo, a rat model and a human cohort of 63 men and 19 women with hand OA and healthy controls, respectively, were used. The baseline circulating OT, interleukin-6, leptin, and oestradiol levels were measured, and hand X-ray examinations were performed for each subject. OT induced increased *aggrecan*, *collagen* (*Col*) *X*, and *cartilage oligomeric matrix protein* mRNA transcript levels in vitro, and the immunolabelling experiments revealed a normalization of Sox9 and Col II protein expression levels. No histological differences in lesion severity were observed between rat OA groups. In the clinical study, a multivariate analysis adjusted for age, body mass index, and leptin levels revealed a significant association between OA and lower levels of OT (odds ratio = 0.77; *p* = 0.012). Serum OT levels are reduced in patients with hand OA, and OT showed a stimulatory effect on chondrogenesis. Thus, OT may contribute to the pathophysiology of OA.

## 1. Introduction

Osteoarthritis (OA) has a complex aetiology that includes genetic, hormonal, metabolic, and biomechanical factors. Specific risk factors such as age, sex, race, trauma, obesity, genetic, and oestrogen and nutritional deficiencies have been identified [1,2,3,4,5]. Oxytocin (OT) is a pituitary hormone that regulates the function of target organs and modulates a wide range of behaviours, such as social recognition, love, and fear [6,7,8,9]. OT receptors are present in the peripheral tissues, including adipose, muscle, subchondral bone, testis, ovary, heart, lung, and vascular tissues [10,11,12,13,14,15,16].

Plasma OT levels were lower in ovariectomized mice and rats than in control animals, and were found to be lower in postmenopausal women who developed osteoporosis than in their healthy counterparts [17]. Daily subcutaneous OT injection prevented and alleviated bone loss in ovariectomized mice by enhancing bone microarchitecture and biomechanical strength and by reducing marrow adiposity [17,18]. Additionally, OT receptor knockout mice developed osteoporosis [19]. OT acts on muscle and subchondral bone, which are important structures in OA physiopathology [20], although the underlying mechanism is not well understood [16]. The subchondral modifications and osteoblast actions in OA are well known [21]. Indeed, our group previously showed that OT can inhibit the differentiation of adipocytes and stimulate that of osteoblasts [17]. Data on the relationship between OT and OA are scarce, and a previous study suggested a protective action of OT on cartilage degradation [16].

This present study investigated the role of OT in OA aetiology. Using two human stem cell lines, we aimed to analyze the role of OT on chondrocyte formation in vitro and explore the effect of OT in a rodent model in vivo. Finally, we analyzed the existence of a correlation, if any, between OT and the development of AO in an animal model and a human cohort.

## 2. Results

### 2.1. Effects of OT on Human Multipotent Adipose-Derived Stem (hMADS) Cell Chondrogenesis

**Figure 1:**
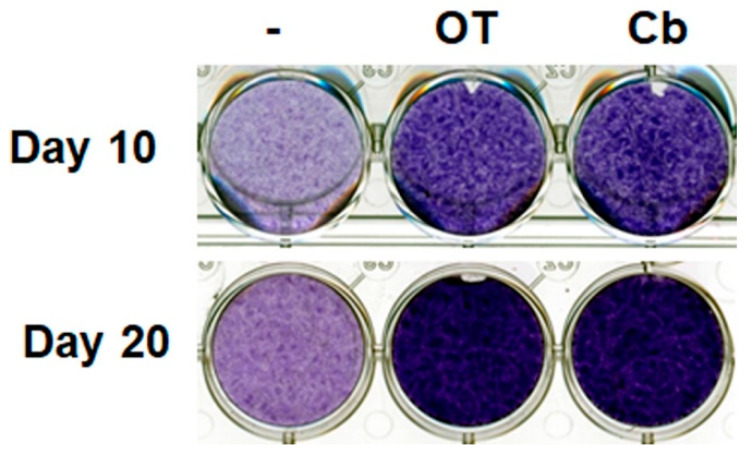
Effects of oxytocin (OT) and carbetocin on extracellular matrix formation. Human multipotent adipose-derived stem (hMADS) cells were seeded in a monolayer in multiwell plates, and then induced to differentiate into chondrocytes by culturing them in differentiation medium for 10 or 20 days in the absence or presence of 30 nM OT or 300 nM carbetocin (Cb). Cells were fixed and stained on the indicated days, and then photographed. Images are representative of three independent experiments.

Undifferentiated hMADS cells and chondrocyte-derived hMADS cells expressed OT receptors (data not shown). Treatment with OT or carbetocin (a stable analogue of OT) increased glycosaminoglycan content in the extracellular environment, as revealed by Toluidine blue staining on days 10 and 20 (Figure 1), indicating the formation of a cartilage matrix. This observation suggests that the activation of the OT pathway can enhance chondrogenesis.

### 2.2. Effects of OT on Chondrogenesis-Related Gene Expression in 2D Cultures of hMADS and hBMS Cells

**Figure 2:**
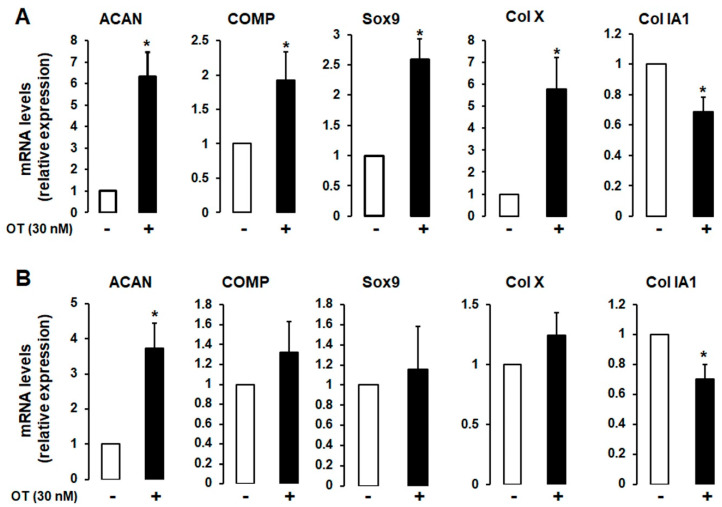
Effect of OT on chondrogenesis in human bone marrow mesenchymal stromal (hBMS) and hMADS cells. hMADS (**A**) and hBMS (**B**) cells were seeded in a monolayer in multiwell plates and induced to differentiate into chondrocytes in a differentiation medium for 21 days in the absence (-) or presence (+) of 30 nM OT. The mRNA transcript levels of *ACAN*, *COMP*, *Sox9*, *Col IA1*, and *Col X* were measured by quantitative reverse transcription-polymerase chain reaction (qRT-PCR). Histograms display mean ± SEM of three independent experiments performed on different series of cells; * *p* < 0.05 vs. untreated cells.

A 2D monolayer cell culture was established to induce chondrogenic differentiation in the absence or presence of 30 nM OT. Differentiated hMADS and human bone marrow mesenchymal stromal (hBMS) cells that expressed chondrogenic markers and OT treatment had similar effects in both cell types (Figure 2A,B), although the mRNA transcript levels of aggrecan (*ACAN*), cartilage oligomeric matrix protein (*COMP*), SRY-related HMG-Box gene 9 (*Sox9)*, and *Col X* were increased to a higher degree in hMADS than in hBMS cells. In contrast, the expression of the fibrous tissue marker *Col IA1* was downregulated by OT treatment (Figure 2).

### 2.3. Effects of OT on hMADS Cell Chondrogenesis in 3D Cultures

**Figure 3:**
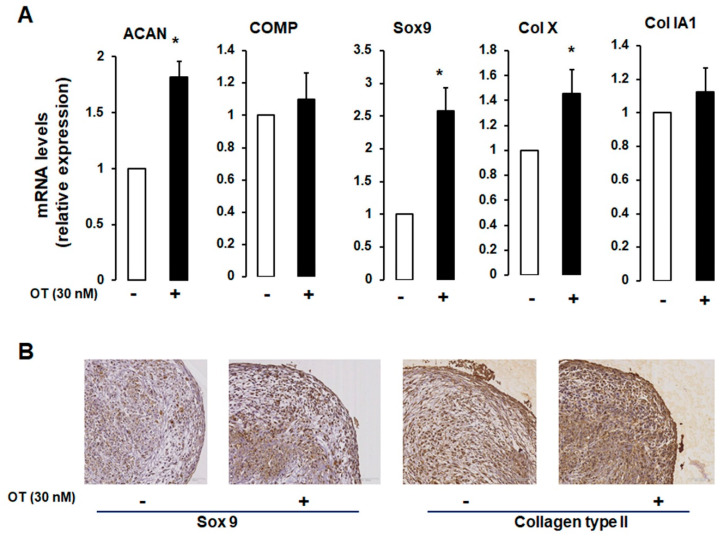
Effect of OT on hMADS chondrogenesis in a 3D culture system. (**A**) Chondrogenic differentiation of hMADS cells was performed in 3D pellet cultures in the absence (-) or presence (+) of 30 nM OT. On day 21, mRNA transcript levels of *ACAN*, *COMP*, *Col Ia1*, and *Col X* were measured by qRT-PCR. Histograms display mean ± SEM data of three independent experiments performed on different series of cells; * *p* < 0.05 vs. untreated cells. (**B**) Examination of pellet sections by haematoxylin-erythrosine-safran staining and indirect immunolabelling using rabbit anti-type IIα collagen and Sox9 antibodies.

We established a 3D cell pellet culture model in which a high cell density leads to compact cell–cell contacts that mimic the in vivo cellular condensation process. As expected, differentiated hMADS cells expressed chondrogenic markers (Figure 3A). OT treatment induced an increase in *ACAN* and *Col X* mRNA expression and a tendency towards increased *COMP* mRNA transcript levels, but there were no changes in the transcript levels of *Col IA1* (Figure 3A).

To further assess the chondrogenic potential of OT, we carried out an immunocytochemical analysis of hMADS cells maintained in pellets that were differentiated for 21 days. Cells cultured in the presence of OT expressed Sox9 and Col II proteins, unlike those cultured without OT (Figure 3B), reflecting the development of a dense filamentous matrix network surrounding the cells. To confirm the effect of OT on hMADS cell differentiation, samples were stained with Alcian blue, Safranin O, and Toluidine blue. Control pellets without OT were small in size and loosely arranged; treatment with OT yielded compact, high-density cultures (data not shown).

### 2.4. Effect of IL1β on Chondrogenic Gene Expression in the Presence of OT

**Figure 4:**
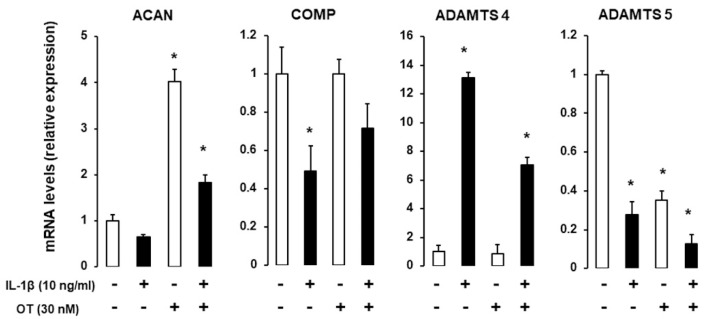
Effect of IL-1β on gene expression in hMADS-derived chondrocytes with or without OT treatment. hMADS cells were induced to differentiate in differentiation medium for 15 days with (+) or without (-) OT; on the last day, cells were exposed to 10 ng/mL IL-1β and mRNA transcript levels of *ACAN*, *COMP*, *Col IA1*, *Col X*, and *ADAMTS-4* and *ADAMTS-5* were analyzed by qRT-PCR. Histograms display mean ± SEM data that are representative of three independent experiments; * *p* < 0.05 vs. untreated cells.

IL-1 is a major proinflammatory cytokine involved in cartilage and bone loss [22]; here, we examined whether OT can reverse the effects of IL-1. hMADS cells were induced to differentiate into chondrocytes in 12-well plates in differentiation medium for 14 days and were exposed on the last day to 10 ng/mL IL-1β. *ACAN* and *COMP* transcripts were downregulated, whereas *Col X* was upregulated in the presence of IL-1β. OT treatment reversed the reduction in *ACAN* expression and boosted the expression of Col X. As expected, IL-1β treatment increased mRNA transcript levels of A disintegrin and metalloproteinase with thrombospondin motifs (*ADAMTS*)-4, which was attenuated by OT treatment (Figure 4).

### 2.5. Animal Study

Rats were subjected to anterior cruciate ligament transection (ACLT) or sham operated and treated with vehicle or OT (1 mg/kg/day) for 28 days. Differences in body weight were observed between the four groups of rats. OT injection inhibited body weight gain in both sham and ACLT rats (Supplementary Figure S1A) as previously reported in mice [23], indicating that OT treatment was effective.

We performed a histological analysis of the medial femur in ACLT rats at 28 days. We evaluated cellularity and surface integrity by haematoxylin-erythrosine-safran (HES) staining, proteoglycan content by Safranin O staining, and collagen content by Sirius red staining (Supplementary Figure S1C). We observed proteoglycan depletion; structural alterations (hypercellularity); fibrosis, hyperplasia, and angiogenesis of the synovial membrane; and bone remodelling and osteolysis in vehicle-treated ACLT rats. Lesion severity was similar in OT-treated ACLT rats. The Mankin’s score revealed significant differences between the two vehicle-treated groups and the two OT-treated groups, with more marked lesions in ACLT rats but no differences when OT was administered (Supplementary Figure S1B).

### 2.6. Human Study Results

We compared serum OT levels in 64 female patients with hand OA and 19 unaffected control subjects. The characteristics of the two groups are shown in Table 1. The mean age was 65 (±11) and 63 (±10) years, respectively; BMI was 24 (±4) and 26 (±5), respectively; and serum OT levels were 1.4 (±2) and 6.5 (±7) pg/mL, respectively. A multivariate analysis adjusted for age, BMI, and leptin levels showed a significant negative association between serum OT levels and hand OA status, with higher OT levels associated with a lower risk of disease (odds ratio = 0.77, *p* = 0.012; Table 2). In the multivariate analysis, there was no relationship between serum OT level and OA severity based on the Verbruggen score (*p* = 0.08), KL score (*p* = 0.22).

## 3. Discussion

OT acts on muscle and subchondral bone, which are important structures in the physiopathology of OA. In this study, we demonstrated a possible association between OT and OA. Our in vitro results show that OT stimulates chondrogenesis through the OT receptor expressed by chondrocytes ([16] and data not shown). We previously reported that OT inhibits the differentiation of adipocytes but induces that of osteoblasts. Moreover, subcutaneous OT administration reversed bone loss in ovariectomized mice—an animal model of menopause—by enhancing bone microarchitecture and biomechanical strength, and reducing marrow adiposity [17,18,24].

The relationship between adipose tissue and OA has been widely studied [25]. Adipose tissue synthesizes and releases adipokines that modulate bone metabolism by directly or indirectly regulating bone formation and resorption. Adipocytes also express OT receptor; signalling through these receptors induces lipolysis [26]. Systemic administration of OT has been shown to affect appetite, body weight gain, glucose homeostasis, and lipid metabolism in animal models [26]. In this present study, we confirmed that OT affects adipose tissue, which was demonstrated by the fact that OT-treated rats gained less body weight than untreated controls.

Oestrogen deficiency plays an important role in the pathophysiology of OA [27,28]. OT promotes osteoblastogenesis in both hMADS and hBMS cells [17,24], which can explain the involvement of OT in OA. During OA development, activation of catabolic enzymes, including matrix metalloproteinases and aggrecanases, leads to softening of the articular cartilage and low-grade inflammation characterized by the release of the cytokines tumour necrosis factor-α and IL-1. In turn, this phenomenon likely further induces the expression of catabolic enzymes, such as ADAMTS-4 [29,30]. We found that OT attenuated the effects of IL-1β, as shown by the reduction in *ADAMTS-4* mRNA transcript levels. Thus, OT may modulate chondrogenesis, at least partially, by abrogating the deleterious effects of IL-1β and attenuating inflammation. Moreover, our in vitro results, which showed the positive effect of OT treatment on various genes involved in chondrogenesis, and those of a recent study showing that OT controls chondrocyte matrix degradation through metalloproteinases [16] strongly support the role of OT in the pathophysiology of OA.

Remodelling of the subchondral bone in OA is mediated by the activities of osteoclasts and osteoblasts [31]. Although the association between subchondral bone and cartilage in OA is not fully understood, several mechanisms have been proposed that include microdamage repair, increased vascularity stimulated by angiogenic factors, and enhanced bone–cartilage crosstalk through an increased number of subchondral plate pores [32]. In animal models, osteoblasts of OT knockout mice exhibit reduced mineralization activity and downregulated expression of genes related to osteoblast differentiation [11]. However, OT stimulates the differentiation of osteoblasts into a mineralizing phenotype by inducing the upregulation of BMP-2, which in turn controls the expression of Schnurri-2 and -3, Osterix, and activating transcription factor-4 [19].

OT has dual effects on osteoclasts, as it is known to stimulate osteoclast formation both directly through the activation of nuclear factor-κB and mitogen-activated protein kinase signalling as well as indirectly through the upregulation of receptor activator of nuclear factor-κΒ ligand [11]. Furthermore, OT inhibits bone resorption by mature osteoclasts by triggering cytosolic Ca^2+^ release and nitric oxide (NO) synthesis [19]. It is therefore possible that in OA, a disease with complex physiopathology that is still not completely elucidated, OT has a beneficial action [10] likely via effects on oestrogens, but also has harmful activities that may include stimulating NO synthesis or upregulating BMP-2. Although BMPs are involved in all phases of chondrogenesis, which affect chondrocyte differentiation and cartilage anabolism [12,13,14], recent studies have shown that BMPs can also have harmful effects on articular cartilage [15].

It is known that there are different osteoarthritic phenotypes and that the risk factors will differ according to the targeted joint, which might explain the lack of effect found in our animal model that may have not been the most suitable. Perhaps a model of metabolic osteoarthritis might have been more appropriate. Alternatively, the duration of rat treatments (28 days) might not have been sufficient, as 8 weeks can be necessary to normalize body weight and osteopenia in mice [17,24]. In our human model, the absence of differences in OT rate according to disease severity may reflect the fact that all of the subjects came from the ADEM cohort, which was focused on severe digital OA. However, the use of subjects who all exhibited advanced osteoarthritis lesions makes it impossible to look for a correlation between the OT rate and severity of osteoarthritis, even though it can show a potential action of OT.

In conclusion, we found that OT stimulates chondrogenesis and that women with digital OA have low circulating levels of this hormone. These findings provide a basis for analysing the mode of action of OT in this disease and may aid in the development of more efficient therapies.

## 4. Materials and Methods

### 4.1. Reagents

Cell culture medium, serum, buffers, and trypsin were purchased from Lonza (Verviers, Belgium), and other cell culture reagents were from Sigma–Aldrich Chimie (Saint-Quentin Fallavier, France). BMP6 and TGF-β3 were from Peprotech (Neuilly-sur-Seine, France).

### 4.2. In Vitro Study

#### 4.2.1. Cell Culture

Human bone marrow mesenchymal stromal (hBMS) cells (Cambrex, Paris, France) and human multipotent adipose-derived stem (hMADS) cells were used for experiments. The establishment and characterization of hMADS cells—including their chondrogenic potential—have been previously described [33,34,35].

Cultures were established as either a two-dimensional (2D) monolayer or 3D pellets. For 2D cultures, hBMS or hMADS cells were seeded in 12-well plates at a density of 2.5 × 10^4^ cells/cm^2^ in Dulbecco’s Modified Eagle’s Medium (DMEM)/low-glucose (1 g/L) supplemented with 10% foetal calf serum, 15 mM HEPES, 2.5 ng/mL human fibroblast growth factor (hFGF-2), 60 μg/mL penicillin, and 50 μg/mL streptomycin (growth medium); hFGF-2 was removed when cells reached confluence and 2 days later (designated as day 0) cells were induced to differentiate by replacing the medium with DMEM/high glucose (4.5 g/L) supplemented with 500 μM ascorbic acid, 10 µg/mL insulin, 5.5 µg/mL transferrin, 5 ng/mL selenium, 1 mM sodium pyruvate, 40 µg/mL l-proline, 100 nM dexamethasone, 10 ng/mL bone morphogenetic protein (BMP)6, and 10 ng/mL transforming growth factor (TGF)-β3 (differentiation medium).

For 3D analysis, hMADS cells were seeded at 5 × 10^5^ cells per 15-mL polypropylene tube and centrifuged for 5 min at 400× *g*. The resultant cell pellet was maintained in growth medium for 3 days, and chondrogenesis was induced with differentiation medium. The lid of the tube was left open to allow for gas exchange. The medium was changed every 3 days, and cells were analyzed on the indicated days. OT (30 nM) was added every day during the differentiation process, and interleukin (IL)-1β was added on day 14.

#### 4.2.2. Real-Time Quantitative Reverse Transcription-Polymerase Chain Reaction (qRT-PCR)

These procedures followed MIQE recommendations [36]. Briefly, total RNA was extracted using the TRI-Reagent kit (Euromedex, Souffelweyersheim, France) according to the manufacturer’s instructions. RNA purity and integrity were evaluated by spectrophotometry on a NanoDrop 1000 instrument (Thermo Fisher Scientific, Waltham, MA, USA) and by SYBR Gold-stained agarose gel electrophoresis (Invitrogen, Carlsbad, CA, USA). Primer sequences designed using Primer Express software (Applied Biosystems, Courtaboeuf, France) are listed in Supplementary Table 1 and were tested for specificity, efficiency, reproducibility, and dynamic range. Amplification was performed in a 10-µL reaction volume with Takara SYBR qPCR Premix ExTaq II (Ozyme, Montigny-le-Bretonneux, France) on a StepOne Plus ABI Real-time PCR instrument (PerkinElmer Life and Analytical Sciences, Boston, MA, USA). The expression levels of selected genes were normalized to those of the *TATA-binding protein* and *36B4* housekeeping genes and quantified using the comparative ΔCt method.

#### 4.2.3. Histological Analysis and Immunohistochemistry

Cell pellets were fixed in phosphate-buffered formaldehyde and then dehydrated through a graded series of ethanol, cleared with xylene, and embedded in paraffin; the block was cut into sections of thickness 5 µm. The sections were stained with HES for morphological analysis, with Alcian blue for proteoglycan detection, and with Toluidine blue to characterize glycosaminoglycans, Safranin O, and other non-neutral polysaccharides. Indirect immunolabelling was performed as previously described [37] using a rabbit anti-type IIα collagen (Col II) antibody (Merck Millipore, Molsheim, France).

At the end of vehicle or OT treatment (28 days after surgery), rats were sacrificed by cervical dislocation under general anaesthesia. Whole joints were dissected, fixed with 4% formalin in 0.1 M phosphate buffer (pH 7.4), and decalcified with Rapide Decalcifiant Osseux (Eurobio-Ingen, Courtaboeuf, France). After embedding in paraffin, 5-µm-thick sections were cut and stained with HES, Toluidine blue, and Sirius red. The severity of OA lesions in the medial part of the femur was graded on a scale adapted from Mankin’s scoring system, which is a 27-point histological/histochemical grading scale that encompasses structure, cellularity, Safranin O staining, thickness of the hypertrophic chondrocyte layer, synovial fibrosis, hyperplasia, angiogenesis, bone remodelling, and osteolysis [23].

For rat animal model studies, see supplementary methods.

### 4.3. Human Study

Study subjects were originally participants in a randomized study between 2010 and 2012 that investigated the efficacy of methotrexate for the treatment of erosive digital OA (ADEM study, supported by the Programme Hospitalier de Recherche Clinique 2007” grant). All subjects provided written informed consent. The local ethics committee “Sud Méditerranée V” approved the cohort study (registered at www.clinicaltrials.gov, No. NCT01068405. 15/02/2011). Selection criteria included pain higher than 4 on a visual analogic scale (0–10); at least three erosive lesions on the proximal interphalangeal (PIP) and/or distal interphalangeal (DIP) joints; and absence of personal or family psoriasis or radiological lesions evoking psoriatic arthritis or other elements that could lead to inflammatory rheumatism. All patients met the American College of Rheumatology classification criteria for hand OA and failed classical treatment [38]. A majority of women were included in the ADEM study; thus, to avoid a sex-related bias, only women were selected in the present analysis. The control group consisted of 19 women of the same age range without osteoporosis, OA, or inflammatory disease who were recruited at the Rheumatology Department of Nice Centre Hospitalier Universitaire.

At baseline, subjects underwent clinical examination, conventional X-ray radiography of the hands, and biological analyses that included measurements of serum OT and leptin levels. Posteroanterior radiographs of both hands were independently scored according to the anatomic phase scoring system [39] by two experienced rheumatologists, and good reliability was obtained (underweighted K = 0.8). Erosive OA was defined as the presence of erosive (E phase) or remodelled radiographic features according to this system in at least three interphalangeal joints. The presence of radiographic carpometacarpal involvement was recorded. Other scoring systems, such as the Kellgren and Lawrence (KL) system, were also used [39,40,41]. Radiological severity was determined based on the KL score by summing the values for PIP (1–5) and DIP (2–5), and the anatomic score with the number of E phases per patient.

### 4.4. Statistical Analysis

Data are expressed as mean values ± SEM and were analyzed using the two-tailed Student’s *t*-test. Differences were considered to be statistically significant at *p* ≤ 0.01. Categorical data are expressed as frequency, and continuous variables are expressed as mean (±SD). The relationship between hand OA and serum OT levels was evaluated with a multivariate logistic regression model adjusted for age in years, body mass index (BMI; continuous variable), and leptin levels, which are known to be potential confounders in analyses of hand OA. The whole study population (i.e., OA patients and controls) was included in the analysis. The relationship between hand OA severity and serum OT levels was also evaluated with a multivariate linear regression model adjusted for the same variables plus IL-6, oestradiol, and adiponectin levels, which are also known confounding factors; control group patients were excluded from this analysis. Two-sided *p* values < 0.05 were considered to denote statistically significant differences between means. Analyses were performed using SAS v.9.1 software (SAS Institute, Cary, NC, USA).

## Figures and Tables

**Table 1:** Characteristics of Patients with and Without Hand Osteoarthritis (OA).

	Patient with OA (*n* = 65)	Patients without OA (*n* = 19)	p*
Mean age (SD), years	64.7 (11)	63.2 (10)	0.26
Mean BMI (SD)	24.3 (4)	25.8 (5)	0.24
Mean oxytocin level (SD), pg/mL	1.4 (2)	6.5 (7)	0.0004
Mean leptin level (SD), ng/mL	17.5 (12)	35.3 (27)	0.004
Mean (SD) of the sum of KL scores	29.8 (14)		
Mean (SD) of the sum of JSN scores	12.5 (5)		

BMI, body mass index; JSN, joint space narrowing; KL, Kellgren and Lawrence; OA, osteoarthritis; p*, *p* value from univariate analysis; SD, standard deviation.

**Table 2:** Factors Influencing Hand Osteoarthritis.

	OR	p*	95% CI
Oxytocin level (pg/mL)	0.77	0.01	(0.65–0.943)
Age	1.04	0.8	(0.98–1.09)
BMI	1.14	0.01	(0.92–1.40)
Leptin level (ng/mL)	0.92	0.0005	(0.86–0.99)

BMI, body mass index; 95% CI, 95% confidence interval; OR, odds ratio; p*, *p* value for logistic regression adjusted for age, BMI, and leptin levels.

## Supplementary Materials

Supplementary materials can be found at https://www.mdpi.com/1422-0067/21/11/3966/s1.

## Abbreviations

ACLT	Anterior cruciate ligament transection
ADAMTS	A disintegrin and metalloproteinase with thrombospondin motifs
BMI	Body mass index
BMP	Bone morphogenetic protein
Cb	Carbetocin
Col	Collagen
COMP	Cartilage oligomeric matrix protein
DIP	Distal interphalangeal
DMEM	Dulbecco’s modified Eagle’s medium
hBMS	Human bone marrow mesenchymal stromal cells
HES	Haematoxylin-erythrosine-safran
hFGF	Human fibroblast growth factor
hMADS	Human multipotent adipose-derived stem
IL	Interleukin
KL	Kellgren and Lawrence
JSN	Joint space narrowing
NO	Nitric oxide
OA	Osteoarthritis
OT	Oxytocin
PIP	Proximal interphalangeal
qRT-PCR	Real-time quantitative reverse transcription-polymerase chain reaction
Sox9	SRY-related HMG-box gene 9
TGF	Transforming growth factor
