# Supplementary material for: Oxytocin Controls Chondrogenesis and Correlates with Osteoarthritis
Source: Int J Mol Sci. 2020 May 31;21(11):3966. doi: 10.3390/ijms21113966 (PMC7312425; doi:10.3390/ijms21113966)
Supplement: Supplementary file 1 [file ijms-21-03966-s001.pdf]

Supplementary materials

Methods

Animal study

All experiments were performed in accordance with international guidelines on the Use of Laboratory Animals and all animal protocols were approved by the ethical animal care committee of the University of Lorraine (CELMEA authorization no. 00061.01). Female Wistar rats (n = 40) weighing 150–180 g (Charles River Laboratories, Lyon, France) were housed in solid-bottomed plastic cages with access to standard laboratory food and water, and were maintained on a 12:12-h light/dark cycle (lights on from 0600–1800) in a controlled temperature chamber. Rats were divided into the following four groups (n = 10 each): 1) sham (daily saline injection); 2) anterior cruciate ligament transection (ACLT) control group (ACLT + daily saline injection); 3) oxytocin (OT) ACLT group (ACLT + injection of 1 mg OT/kg/day); and 4) sham OT group (injection of 1 mg OT/kg/day).

ACLT model

On day 0, rats underwent ACLT {Galois, 2004 #4071} under anaesthesia (intraperitoneal injection of a mixture of 38 mg/kg ketamine and 1 mg/kg acepromazine). A parapatellar skin incision was made first on the medial side of the right knee joint, and then on the medial side of the patellar tendon. The patella was laterally dislocated to access the joint space and the ACL was transected in the flexed knee position. A positive anterior drawer test confirmed complete transection of the ligament. The joint was irrigated with sterile saline to prevent ancillary inflammation, and the wound was sutured closed.

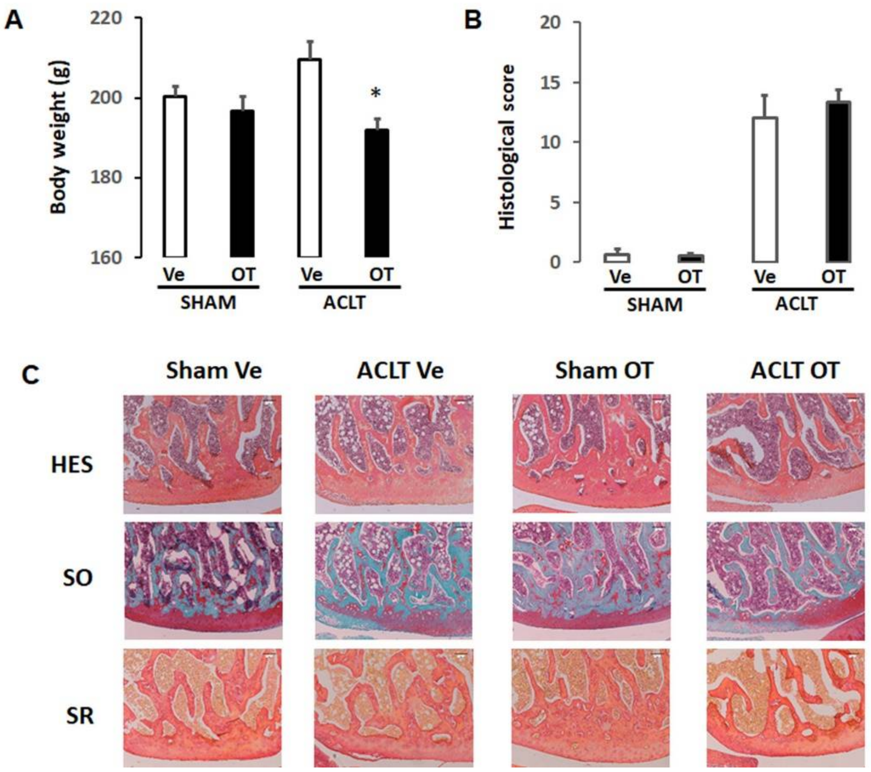

Supplementary Figure 1. Effects of OT on cartilage injury. A–B: Body weight (**A**) and histological analysis of medial femur and histological score (**B**) in sham and ACLT rats treated with OT of vehicle (Ve) for 28 days. (**C**) Determination of proteoglycan and collagen contents by Safranin O (SO) and Sirius red (SR) staining, respectively. Magnification: 10×; $*p < 0.05$ vs. untreated rats.

Supplementary Table 1. Sequences of primers for gene expression analysis

Gene	Complete name	Forward primer	Reverse primer
Col IA1	Collagen type I, alpha1	ACCTGCGTGTACCCCACTCA	CCGCCATACTCGAACTGGAA
Col X	Collagen type X	AGGAATGCCTGTGTCTGCTT	ACAGGCCTACCCAAACATGA
ACAN	Aggrecan	TCGAGGACAGCGAGGCC	AGGGTGTAGCGTGTAGAGA
COMP	Cartilage oligomeric matrix protein	CAATGAACAGCGACCCAG G	TCACATGGAACGTGCCCTC
ADAMTS-4	A disintegrin and metalloproteinase with thrombospondin motifs 4	CCCCAGACCCCGAAGAGCCA	CCCGCTGCCAGGCACAGAAG
ADAMTS-5	A disintegrin and metalloproteinase with thrombospondin motifs 5	TATGACAAGTGCGGAGTATG	TTCAGGGCTAAATAGGCAGT
Sox 9	(Sex determining region Y)-box 9	CAGTACCCGCACTTGCACAAC	ACTTGTAATCCGGGTGGTCCTT
TBP	TATA-binding protein	CACGAACCACGGCACTGATT	TTTTCTTGCTGCCAGTCTGGAC
36B4	60S Acidic ribosomal protein P0	TGCATCAGTACCCCATTCTATCAT	AGGCAGATGGATCAGCCAAGA
